# Editorial: Safety consideration in the development of anti-tumor monoclonal antibodies during drug development

**DOI:** 10.3389/fonc.2025.1640377

**Published:** 2025-07-17

**Authors:** Tao Wang, Shaad Essa Abedin, Jan Willem van der Laan

**Affiliations:** ^1^ Office of Pharmaceutical Quality Research, Office of Pharmaceutical Quality, Center for Drug Evaluation and Research, Food and Drug Administration, Silver Spring, MD, United States; ^2^ Clinical Development, iTeos Therapeutics, Watertown, MA, United States; ^3^ Leiden Academic Centre for Drug Research, Division of Cell Systems and Drug Safety, Leiden University, Leiden, Netherlands; ^4^ Retired, Ede, Netherlands

**Keywords:** drug development, monoclonal antibody drugs, bispecific antibodies, antibody-drug conjugates, anti-tumor therapy, chemistry, manufacturing, and controls (CMC), toxicology

Monoclonal antibody (mAb) therapy has changed the landscape of anti-cancer treatment and has become a standard of care for many types of cancer. While achieving effective anti-cancer activity is essential, ensuring quality and safety is equally important during the development of a successful antibody-based anticancer therapy. From a total of ten submissions received for this Research Topic, seven articles were selected for publication. As editors for this Research Topic, we appreciate the contributions of all authors and reviewers of these excellent articles in the field of antibody safety. The accepted articles in this Research Topic cover many aspects of the development of safe and efficacious antibody drugs, including one comprehensive review paper on the challenges of the development of such antibodies, a research paper on the potential impact of Fab (fragment antigen-binding) region glycosylation on antigen binding and the effector function of the Fc (Fragment crystallizable) region, as well as five papers that focus on safety issues from a clinical perspective.

The review article by Shapiro, an internationally acclaimed CMC (Chemistry, manufacturing, and controls) assessor and scientist, provides a comprehensive overview of several critical aspects of mAb drug development and quality. Her review focuses on challenges and strategies in the development of “patient-centric” CMC development strategy and other regulatory aspects to ensure the quality, safety, and efficacy of antibodies including new formats of mAb drugs such as bispecific antibodies and antibody-drug conjugates. Moreover, it highlights the importance of understanding the mechanisms of actions, structure, and biology of therapeutic mAb drugs, which is key to developing a successful anti-cancer therapeutic antibody program. Altogether, this review is highly informative for investigators in the field of therapeutic antibody development.

A research paper by Saporiti et al. discussed the glycosylation in the Fab region occurring in many therapeutic antibodies including cetuximab, an approved recombinant chimeric human/mouse IgG1 mAb against the epidermal growth factor receptor (EGFR) for the treatment of some types of cancer. Fab region glycosylation has been shown to impact antibody stability, binding ability to the antigen, and the Fc effector functions. The authors suggest that the Fab region glycosylation might result in a conformation change of cetuximab, demonstrated by an *in silico* computational simulation model, and that this conformation change by Fab region glycosylation might affect antigen binding and Fc effector function such as antibody dependent cellular cytotoxicity (ADCC). Although the conclusion of this study needs to be further investigated and verified, the computational simulation approach may provide a valuable tool to understanding the potential impacts of Fab region glycosylation on antibody functions.

Changing the schedule (not the route) of administration can have also impact occurrence of adverse effects, as shown in a paper by Varo et al., in this Research Topic. The authors have shown that reducing the starting dose in a “Step-UP” infusion regimen reduces severe adverse effects compared to a Standard Infusion regimen (SIR), substantially by lowering the severe hypotension and bronchospasm occurring with a high frequency after SIR. It was remarkable that such a change in dosing schedule had such important effects on medical significant adverse effects, often leading to hospitalization.

Liu et al. contributed to this Research Topic by presenting a real-world pharmacovigilance study of the first FDA-approved drug-conjugate polatuzumab vedotin. The authors have used the FDA Adverse Event Reporting System (FAERS) to provide data for their analysis. Adverse effects related with death cases have been reported in this study with a recommendation to closely monitor these in order to prevent the occurrence of malignant adverse effects that threaten patient safety.

Li et al. analyzed the FAERS database demonstrating that the addition of pembrolizumab to paclitaxel in the treatment of breast cancer leads to immune-mediated toxicities, including adrenal insufficiency, myocarditis, hypophysitis, and enterocolitis, while also identifying their median onset times. These real-world safety signals refine the therapeutic risk-benefit assessment observed in clinical trials, emphasizing the need for early clinical surveillance when implementing such a promising chemo-immunotherapy combination.

The Chen et al. case series on bevacizumab-associated cerebrovascular accidents serves as a timely warning that anti-VEGF antibodies can provoke late, organ-specific harm. By pairing real-world analysis of FAERS data with clinical imaging, the authors uncover stroke and hemorrhage signals that could be obscured in trials and demonstrate that simple schedule adjustments may reduce risk. Their work enriches this Research Topic by translating pharmacovigilance data into concrete bedside guidance and reinforces the need for neurosafety monitoring in mAb programs.

In conclusion, this Research Topic highlights three interconnected priorities for the field of antibody safety. First, a molecular-level understanding of antibody structure, from Fab glycosylation to drug-conjugate linkers, remains foundational for anticipating off-target biology and its potential clinical impacts. Second, adaptive CMC and dosing strategies, such as step-up infusions, demonstrate that thoughtful engineering of both product and regimen can significantly reduce toxicity without compromising efficacy. Finally, systematic mining of global pharmacovigilance resources provides critical “early-warning signs” that complement controlled trials and guide real-world risk mitigation. By triangulating insights from bench chemistry, quality, clinical pharmacology, and post-marketing surveillance, the contributors illuminate a pragmatic path toward genuinely patient-centric development of therapeutic antibodies. We hope that this Research Topic stimulates deeper cross-disciplinary dialogue and accelerates the translation of safety intelligence into next-generation antibody platforms that deliver lasting benefits with uncompromised safety for cancer patients worldwide.

